# Endotyping Insulin–Glucose Homeostasis in Hidradenitis Suppurativa: The Impact of Diabetes Mellitus and Inflammation

**DOI:** 10.3390/jcm14072145

**Published:** 2025-03-21

**Authors:** Nessr Abu Rached, Johannes W. Dietrich, Lennart Ocker, Eggert Stockfleth, Yannik Haven, Daniel Myszkowski, Falk G. Bechara

**Affiliations:** 1 International Centre for Hidradenitis Suppurativa/Acne Inversa (ICH), Department of Dermatology, Venereology and Allergology, Ruhr-University Bochum, 44791 Bochum, Germany; lennart.ocker@kklbo.de (L.O.); eggert.stockfleth@kklbo.de (E.S.); yannik.haven@kklbo.de (Y.H.); daniel.myszkowski@kklbo.de (D.M.); falk.bechara@kklbo.de (F.G.B.); 2 Skin Cancer Center, Department of Dermatology, Venereology and Allergology, Ruhr-University Bochum, 44791 Bochum, Germany; 3 Diabetes, Endocrinology and Metabolism Section, Department of Internal Medicine I, St. Josef Hospital, Ruhr University Bochum, Gudrunstr. 56, 44791 Bochum, Germany; johannes.dietrich@kklbo.de; 4 Diabetes Centre Bochum-Hattingen, St. Elisabeth-Hospital Blankenstein, Im Vogelsang 5-11, 45527 Hattingen, Germany; 5 Centre for Rare Endocrine Diseases, Ruhr Centre for Rare Diseases (CeSER), Ruhr University Bochum and Witten/Herdecke University, Alexandrinenstr. 5, 44791 Bochum, Germany; 6 Centre for Diabetes Technology, Catholic Hospitals Bochum, Gudrunstr. 56, 44791 Bochum, Germany

**Keywords:** insulin–glucose homeostasis, HOMA-IR, hidradenitis suppurativa, HS, dermato-endocrinology, insulin resistance

## Abstract

**Background:** Hidradenitis suppurativa (HS) is a chronic inflammatory skin disease often associated with metabolic disorders such as diabetes mellitus. Recent research suggests a link between systemic inflammation and insulin–glucose dysregulation in HS. This study investigates the relationship between insulin–glucose homeostasis, diabetes mellitus and the haptoglobin concentration in HS patients. **Methods:** We assessed 95 HS patients and 49 controls using validated fasting-based function tests, including the Structural Parameter Inference Approach (SPINA), Homeostasis Model Assessment (HOMA) and Quantitative Insulin Sensitivity Check Index (QUICKI). **Results:** The HS patients had a significantly higher fasting insulin concentration (97.2 vs. 69.0 pmol/L, *p* = 0.035), increased insulin resistance (HOMA-IR: 3.47 vs. 2.57, *p* = 0.016) and impaired insulin sensitivity (SPINA-GR: 1.34 vs. 1.76 mol/s, *p* = 0.017). In diabetes, the insulin sensitivity was more strongly reduced (SPINA-GR: 0.61 vs. 1.41 mol/s, *p* = 0.0057) and the insulin resistance increased (HOMA-IR: 7.3 vs. 3.2, *p* = 0.017). Higher haptoglobin concentrations were accompanied by worse glycaemic control, demonstrating a significantly elevated fasting glucose (5.77 vs. 5.11 mmol/L, *p* = 0.043) concentration and HbA1c (5.7% vs. 5.4%, *p* = 0.0081) fraction. **Conclusions:** Our findings suggest that chronic inflammation in HS contributes to metabolic dysregulation, worsening insulin resistance and glycaemic control, particularly in those with elevated haptoglobin or diabetes.

## 1. Introduction

Hidradenitis suppurativa (HS) is a chronic inflammatory disease affecting the skin [1]. The disease has a high prevalence, affecting more than 2% of the population in industrialised countries. Typically, recurrent inflammatory nodules, abscesses, scars and fistulae develop in the intertriginous areas of the skin, which are particularly exposed to pressure [2,3]. The pathogenesis of HS is multifactorial and is considered to include genetic, immunological, microbial and hormonal environmental factors [4,5,6,7,8,9]. Treatment of HS involves a multimodal approach with local therapy, surgery, supportive therapy, antibiotics and biologics. Recently, a third biologic, bimekizumab, was approved by the EMA for moderate to severe HS, following secukinumab and adalimumab [10].

Recently, increasing evidence has been found that the inflammatory state is not limited to the skin organ but also manifests systemically [11,12]. In addition to dermatological symptoms, affected patients often suffer from systemic comorbidities, particularly metabolic disorders, including obesity and diabetes mellitus [13,14,15,16]. In HS patients, inflammatory markers such as haptoglobin are associated with disease severity and metabolic risk [17]. The role of meta-inflammation, a chronic, low-grade inflammation associated with metabolic disorders [18,19], is poorly understood in the context of HS. However, potential therapeutic benefits of targeting meta-inflammation are already being discussed [20]. Interestingly, optimised anti-inflammatory treatment resulted in the rapid remission of diabetes mellitus, in parallel to reduced systemic inflammation and HS symptoms [21]. The exact mechanism of diabetes remission in this setting is not yet clear, but we suspect a potential link between inflammation and metabolism. Eliminating chronic inflammation may have a positive effect on the stability and control of metabolism [22,23]. Additional research into insulin–glucose homeostasis is needed to better understand this exact relationship and to open up avenues for potential therapeutic interventions.

Physiological endotyping is a promising approach that has paved the way to a better understanding of metabolic disorders, including diabetes mellitus, by defining clusters of health conditions marked by distinct functional mechanisms [24,25]. Up to now, however, the possible mechanisms of altered insulin–glucose homeostasis in patients with HS have not been investigated on the level of physiological endotypes. To fill this gap, we observed two cohorts of persons with and without HS and performed in vivo calculations of validated structural parameters of insulin–glucose feedback control from measured fasting concentrations of hormones or metabolites, including the Structural Parameter Inference Approach for carbohydrate homeostasis (SPINA Carb), Quantitative Insulin Sensitivity Check Index (QUICKI), and Homeostasis Model Assessment (HOMA). The HOMA and QUICKI parameters are established calculated biomarkers for insulin–glucose homeostasis, whereas several studies showed the SPINA parameters to have higher accuracy, reliability and diagnostic utility [26,27]. On this basis, the present work pursues two goals: (1) to characterise the diabetes subgroups in HS based on the foundations of the ANDIS clustering [28] approach and (2) to investigate the interaction between insulin–glucose homeostasis, diabetes mellitus and the haptoglobin concentrations in patients with inflammatory processes that interact and determine to what extent they affect the clinical pattern of HS.

## 2. Materials and Methods

### 2.1. Design and Setting

This monocentric investigation collected data on insulin–glucose homeostasis in 95 patients with HS from the International Centre for Hidradenitis suppurativa/Acne inversa Bochum, as well as in 49 control persons without HS but with the same age and sex distribution as the HS group. Two experienced dermatologists independently confirmed the diagnosis of HS. In cases of discrepancies, another experienced dermatologist was consulted. All patients without complete data were excluded from this study.

Screening for diabetes mellitus was performed according to the guidelines of the German Diabetes Society [29] and the American Diabetes Association [30]. The patients underwent blood sampling after an overnight fast of 8 to 12 h. A patient was classified as having diabetes if the HbA1c fraction exceeded 6.5% or if the fasting glucose concentration was ≥126 mg/dL (7.0 mmol/L). Sub-classification according to the ANDIS clustering was performed with a simplified approach based on the age at diagnosis of diabetes, body mass index (BMI) and pancreatic beta-cell antibody titres, as previously described [31].

### 2.2. Sample Size Calculation

Assuming a two-sided significance level of 0.05 and a power of 90% (1 − β), we accounted for the expected probabilities of the event in the case group (43.4%) and the control group (16.4%) [32]. The expected event data used to calculate the sample size were obtained from another case-control study of HS patients [32]. According to the study design, a case-control ratio of 2:1 was used. The calculations were performed using established formulas for matched case-control studies, considering the correlation between matched pairs. The calculated sample size required at least 62 cases and 31 controls. The calculation of the sample size was based on Wang et al. [33], which is available at https://riskcalc.org/samplesize/ (accessed on 1 June 2023).

### 2.3. Physiological Calculations and Cut-Off Value

In order to obtain information on the endotype of insulin–glucose homeostasis, the secretory capacity of pancreatic beta cells (SPINA-GBeta), the insulin receptor gain (SPINA-GR as a biomarker for insulin sensitivity) and the static disposition index (SPINA-DI, a marker for the loop gain of the homeostatic system) were calculated as previously described [26,27].

For comparison, additional calculated biomarkers for beta-cell function and insulin sensitivity were determined as the HOMA-Beta, HOMA-IR and HOMA-IS indices [34], and as the QUICKI approach [35].

The haptoglobin concentration was classified as low or high according to a predefined cut-off value of 209.8 mg/dL [17]. Moreover, 5 mg/dL was selected as the cut-off value for c-reactive protein.

### 2.4. Statistical Analysis

Data are presented as the mean ± standard error of the mean for the Gaussian distributed variables, as the median with interquartile range (IQR) for the continuous variables with a non-normal distribution, and as the frequency (%) for categorical variables. A normal distribution was assessed using the Kolmogorov–Smirnov test and Q–Q plots, and the homogeneity of variances with an F-test. Where the conditions for a *t*-test were not met, the U-test according to Wilcoxon, Mann and Whitney was used to compare continuous variables between groups. The categorical variables were compared using the Chi-squared test.

The statistical analysis was performed using custom S scripts for R on macOS, version 4.4.0, with the packages Hmisc, aod, car, ellipse, ordinal, visreg and fmsb. A two-tailed *p* < 0.05 was considered to be statistically significant. All *p*-values ≥ 0.0001 are reported with two significant digits.

## 3. Results

### 3.1. Patient Characteristics

In total, we included 95 patients with HS and 49 control persons with the same sex and age ranges. Detailed characteristics of the two cohorts are reported in Table 1. There was no significant difference in gender (39% female in HS group vs. 43% in controls, *p* = 0.78) or age (median 41 vs. 40 years, *p* = 0.54). However, the HS group had a significantly higher BMI (median 31.1 vs. 27.4 kg/m^2^, *p* = 0.0003).

### 3.2. Prevalence of and Therapy for Diabetes

Diabetes mellitus was more common in the subjects with HS (18%) than in the control persons (4%, *p* = 0.0039). Unlike the controls, the majority of HS patients with diabetes were affected by the mild obesity-related diabetes (MOD) cluster (Table 1).

In the HS group, ten patients with diabetes were treated with metformin (59% of all the diabetic persons) and three persons with sitagliptin (18%). In the control group, two received metformin (100% among the patients with diabetes) and one sitagliptin (50%). One person, respectively, was treated with vildagliptin, saxagliptin, glimepiride and empagliflocin in the HS group and no person in the control group.

### 3.3. Measured and Calculated Biomarkers for Insulin–Glucose Homeostasis

**Figure 1:**
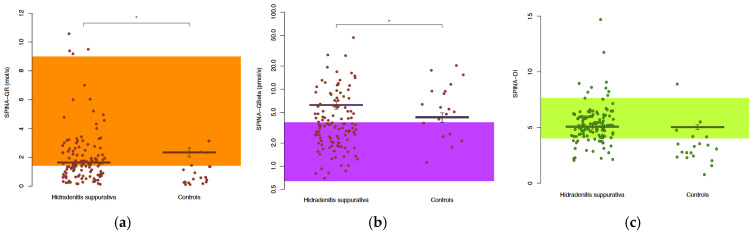
Compared to normal controls, insulin sensitivity is more heterogeneous but, on average, lower in patients with HS and partly compensated for by increased pancreatic beta-cell function: calculated biomarkers for insulin sensitivity (SPINA-GR, (**a**)), pancreatic beta-cell function (SPINA-GBeta, (**b**)) and loop gain (disposition index, SPINA-DI, (**c**)). Shaded areas denote the reference ranges for the respective parameters in a healthy population with a normal BMI; * significant result.

The biomarkers for insulin–glucose homeostasis were assessed in the patients with HS and the controls (Table 2 and Figure 1). The fasting glucose concentrations were similar between the two groups (5.1 vs. 4.9 mmol/L, *p* = 0.35), but the fasting insulin levels were significantly higher in the HS patients (97.2 vs. 69.0 pmol/L, *p* = 0.035). The HS patients also had elevated SPINA-GBeta (3.87 vs. 2.82 pmol/s, *p* = 0.041) and lower SPINA-GR (1.34 vs. 1.76 mol/s, *p* = 0.017), indicating impaired insulin sensitivity with compensatory beta-cell activation. Furthermore, the insulin resistance as measured by HOMA-IR was significantly higher in the HS group (3.47 vs. 2.57, *p* = 0.016), and the insulin sensitivity as measured by HOMA-IS and QUICKI was significantly reduced in the HS patients (both *p* = 0.016). SPINA-DI, which indicates that the loop gain of the homeostatic system was similar between both groups, suggesting a state of dynamic compensation, but lower in the patients receiving treatment.

### 3.4. Parameters of Insulin–Glucose Homeostasis in Patients with HS and with and Without Diabetes Mellitus

**Figure 2:**
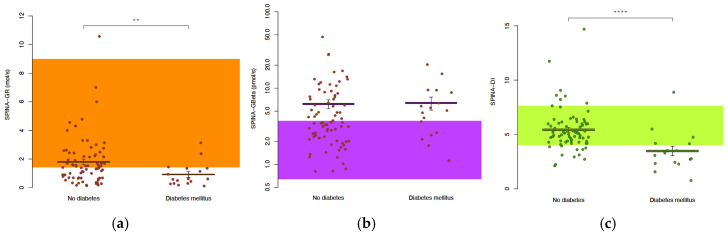
In patients with HS and diabetes, insulin sensitivity is reduced (**a**) without dynamic compensation via pancreatic beta-cell function (**b**), resulting in a reduced loop gain of the feedback control system (**c**). See the legend of Figure 1 for additional explanations; **, *p* < 0.01; ****, *p* < 0.001.

Among the patients with HS, those with diabetes mellitus (n = 17) showed significantly altered insulin–glucose homeostasis compared to those without diabetes (n = 78). The fasting glucose was significantly higher in the diabetes group (6.28 vs. 5.03 mmol/L, *p* < 0.0001; Table 3 and Figure 2), as was the HbA1c (6.8% vs. 5.4%, *p* < 0.0001), reflecting poorer glycaemic control. While the fasting insulin concentrations were elevated in the diabetes group (145.8 vs. 86.4 pmol/L), this difference was not statistically significant (*p* = 0.13). SPINA-GR, an indicator of insulin sensitivity, was significantly lower in the diabetes group (0.61 vs. 1.41 mol/s, *p* = 0.0057), and SPINA-DI was significantly reduced in the diabetes group (3.29 vs. 5.11, *p* < 0.0001). The markers of insulin resistance (HOMA-IR) were higher in the diabetic patients (7.3 vs. 3.2, *p* = 0.017), while the insulin sensitivity (HOMA-IS and QUICKI) was significantly lower (both *p* = 0.017).

### 3.5. Parameters of Insulin–Glucose Homeostasis in Patients with HS and Different Expressions of Haptoglobin Concentration

**Figure 3:**
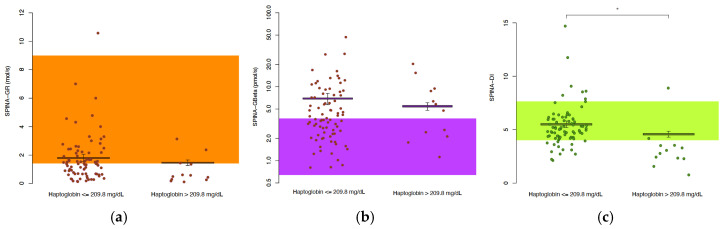
In persons with HS and a high haptoglobin concentration, insulin sensitivity and beta-cell function are similar but combine to provide a reduced disposition index. See the legend of Figure 1 for additional explanations.

**Figure 4:**
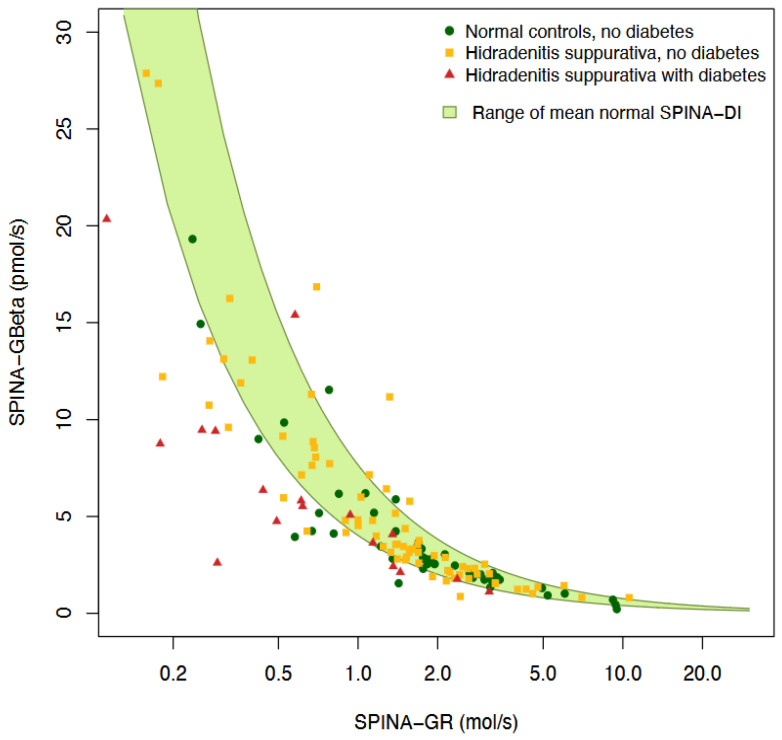
The majority of patients with HS and diabetes are faced with rather low insulin sensitivity and a lack of dynamic compensation via pancreatic beta-cell function, resulting in a reduced disposition index. Plot of SPINA-GBeta versus SPINA-GR in patients with HS and controls.

Table 4 and Figure 3 compare the insulin–glucose homeostasis parameters in the patients with hidradenitis suppurativa (HS) with lower (≤ 209.8 mg/dL, n = 51) and higher (> 209.8 mg/dL, n = 44) haptoglobin concentrations. The patients with higher haptoglobin levels had significantly elevated fasting glucose (5.77 vs. 5.11 mmol/L, *p* = 0.043) and HbA1c (5.7% vs. 5.4%, *p* = 0.0081), indicating poorer glucose regulation. However, there was no significant difference in the fasting insulin concentration (100.2 vs. 90.0 pmol/L, *p* = 0.68) between the two groups. SPINA-DI, a marker of the loop gain, was significantly lower in the higher haptoglobin group (4.37 vs. 5.11, *p* = 0.028), suggesting a reduced compensation via β-cell function. No significant differences were observed in the other parameters, such as SPINA-GBeta, SPINA-GR, HOMA-Beta, HOMA-IR, HOMA-IS and QUICKI, indicating similar insulin sensitivity and resistance profiles between the groups. The haptoglobin concentration was higher in the patients receiving antidiabetic medication (Table 5). Figure 4 shows the relation between beta-cell function (SPINA-GBeta) and insulin sensitivity (SPINA-GR) in the patients with HS and the controls, along with the zone of a normal disposition index.

## 4. Discussion

In this study, we analysed the endotypes of diabetes mellitus in hidradenitis suppurativa. The main findings were that (1) diabetes is more common in HS than in the general population and a control group with the same age and sex distribution; (2) most diabetic patients with HS belong to the MOD cluster of type 2 diabetes; (3) insulin sensitivity is lower in HS compared to controls, together with compensatorily increased beta-cell function; (4) if HS is accompanied by diabetes, the compensation via beta-cell function is insufficient, leading to a lower disposition index and impaired glucose metabolism; and (5) the overall glucose homeostasis is especially deficient in patients with elevated haptoglobin concentration.

A comparatively high prevalence of diabetes was previously described in HS and associated with more severe forms of the disease [31,36]. Several other studies observed HS to be accompanied by insulin resistance [37,38]. This finding could be confirmed in the present study using several methods. Accordingly, we found subtypes of type 2 diabetes with leading insulin resistance (MOD and SIRD) to be more common than subtypes with leading beta-cell dysfunction (SIDD and MARD) [31]. Au contraire, in the majority of patients, the beta-cell function was increased to compensate for the insulin resistance. This observation confirms the theory of dynamical compensation, which predicts an increasing gland mass if the endocrine organs are stimulated by signals of continued demand [39,40,41]. The insufficiency of this form of compensation marks the transition from simple obesity to diabetes [26]. This is confirmed in the present study by the reduced static disposition index (SPINA-DI) in the diabetic patients with HS.

Interestingly, patients with HS who received antidiabetic agents had higher HbA1c fractions, lower SPINA-GR and lower SPINA-DI than untreated persons. In an interventional experiment, one would expect an effect in the opposite direction, i.e., lower HbA1c and higher SPINA-GR and SPINA-DI. Our findings may result from the observational design of our study and a selection effect, where treated patients represent a more severe phenotype of diabetes mellitus. Accordingly, the patients treated with antidiabetic medication had higher Hurley scores and haptoglobin concentrations than the untreated persons.

Meta-inflammation may be the key mechanism linking HS to insulin resistance and, in a subgroup, beta-cell failure. The use of anti-IL-17 or anti-TNF-α biologics, GLP-1 receptor agonists and therapies to reduce meta-inflammation could improve insulin sensitivity. This hypothesis is speculative at this stage and needs to be validated by further prospective studies. Immunoactivation, resulting from both overnutrition and inflammatory disease, has been associated with cardiometabolic syndromes [42]. Minhoff et al. showed in an analysis that metabolically unhealthy overweight/obesity is an independent risk factor for HS, and interestingly, normal weight individuals with HS are more likely to be metabolically unhealthy than controls [37]. Dysregulated adipokine release with a reduced adiponectin concentration and increased release of visfatin, leptin, resistin and retinol-binding protein 4 from adipocytes, as well as a pro-inflammatory cytokine pattern including increased concentrations of TNF α, IL-1β and IL-17, promote insulin resistance and other hallmarks of metabolic syndrome [7]. On the other hand, decreased autophagy, glucotoxicity and lipotoxicity as consequences of insulin resistance facilitate systemic inflammation and visceral obesity [43]. Since similar mechanisms apply to dermal inflammation in HS, it may be assumed that a positive feedback loop between inflammation and metabolic derailment maintains the chronicity of the disease [44]. The observation that the SPINA-DI is reduced and that the fasting glucose concentration and HbA1c fraction are increased in subjects with elevated concentrations of haptoglobin, an acute-phase protein, supports this hypothesis. It implies that patients affected by HS might benefit from screening for cardiometabolic risk factors [45] and an integrated therapeutic approach, addressing both insulin resistance and inflammation [46,47,48,49,50]. A possible link between meta-inflammation and metabolic syndrome in HS could be the PI3K/AKT pathway, which plays an important role in obesity and type 2 diabetes [51]. An imbalance can lead to the development of obesity and type 2 diabetes mellitus [51]. In HS, the imbalance could be caused by chronic inflammation and an imbalance between pro- and anti-inflammatory cytokines. In particular, increased levels of cytokines such as TNF-α, IL-1β and IL-17 are present in both HS and metabolic disorders, supporting this hypothesis [52,53].

The strengths of this study include the use of validated calculated biomarkers for the endotypes of insulin–glucose homeostasis, the stratification for the severity of inflammation and the comparison with a group of unaffected persons without HS and/or diabetes. A limitation of this study results from its observational design bringing along potential undetected confounders. Another limitation is that we could only use static, fasting-based function testing. Additional information may be obtained by dynamical testing, e.g., with glucose tolerance tests and glucose clamp investigations, which should be addressed in future studies.

In particular, future studies should also investigate the aspartate transaminase-to-platelet ratio index (APRI), a biomarker for hepatic fibrosis in metabolic dysfunction-associated steatotic liver disease (MASLD), as previously shown in stage I and II melanoma [54]. Performing future studies in a multi-centre setting could help to raise the statistical power in order to detect mechanisms with lower effect size.

## 5. Conclusions

In summary, the patients with HS showed significantly higher fasting insulin levels, increased insulin resistance (HOMA-IR), and reduced insulin sensitivity (SPINA-GR) compared to the controls. Additionally, those with an elevated haptoglobin concentration had significantly higher fasting glucose and HbA1c, indicating impaired glucose regulation. Chronic inflammation in hidradenitis suppurativa (HS) is linked to significant insulin–glucose dysregulation, particularly in patients with a higher degree of inflammation.

## Figures and Tables

**Table 1:** Basic characteristics of the study population in the two cohorts. Data are reported as the count (percentage) and median (1st–3rd quartile).

Parameter	Hidradenitis Suppurativa (n = 95)	Controls (n = 49)	*p*-Value
Sex			0.78
Female (%)	37 (39%)	21 (43%)	
Male (%)	58 (61%)	28 (57%)	
Age (years)	41 (33–52)	40 (34–46)	0.54
BMI (kg/m^2^)	31.1 (27.2–36.2)	27.4 (22.7–30.8)	0.0003 *
Diabetes mellitus (%)	17 (18%)	2 (4%)	0.039 *
MOD (%)	12 (13%)	0 (0%)	
SIRD (%)	3 (3%)	1 (2%)	
SIDD (%)	1 (1%)	1 (2%)	
MARD (%)	1 (1%)	0 (0%)	
Antidiabetic medication			
Metformin (%)	10 (11%)	2 (4%)	0.31
Sitagliptin (%)	3 (3%)	1 (2%)	1.00
Vildagliptin (%)	1 (1%)	0 (0%)	1.00
Saxagliptin (%)	1 (1%)	0 (0%)	1.00
Glimepiride (%)	1 (1%)	0 (0%)	1.00
Empagliflocin (%)	1 (1%)	0 (0%)	1.00
Hurley stage	2 (2–3)	N/A	N/A
SAHS scale	7 (5–9)	0 (0–0)	<0.0001 *
Number of exacerbations	0 (0–2)	0 (0–0)	<0.0001 *
Affected regions	3 (2–5)	0 (0–0)	<0.0001 *
Fistulae	4 (2–8)	0 (0–0)	<0.0001 *
Nodules	2 (1–4.5)	0 (0–0)	<0.0001 *
Abscesses	0 (0–0)	0 (0–0)	0.0098 *

* significant result; N/A, not applicable.

**Table 2:** Measured and calculated biomarkers for insulin–glucose homeostasis in patients with HS and controls.

Parameter (Reference Range)	Hidradenitis Suppurativa (n = 95)	Controls (n = 49)	*p*-Value
Fasting glucose (mmol/L)	5.1 {4.6–5.9)	4.9 (4.6–5.6)	0.35
Fasting insulin (pmol/L)	97.2 (53.8–189.6)	69.0 (46.0–121.2)	0.035 *
HbA1c (%)	5.6 (5.3–6.0)	N/A	N/A
SPINA-GBeta (0.64–3.73 pmol/s)	3.87 (2.32–7.81)	2.82 (1.86–4.25)	0.041 *
SPINA-GR (1.41–9.00 mol/s)	1.34 (0.62–2.16)	1.76 (1.06–2.90)	0.017 *
SPINA-DI (4.01–7.65)	4.82 (4.04–5.96)	5.17 (4.09–5.95)	0.51
HOMA-Beta	197.1 (119.3–401.1)	156.5 (117.6–262.1)	0.14
HOMA-IR (<2.5)	3.47 (2.03–7.70)	2.57 (1.57–4.56)	0.016 *
HOMA-IS (>0.4)	0.29 (0.13–0.49)	0.39 (0.22–0.64)	0.016 *
QUICKI (>0.4)	0.32 (0.29–0.34}	0.33 (0.31–0.36)	0.016 *

* significant result; N/A, not applicable.

**Table 3:** Parameters of insulin–glucose homeostasis in patients with HS and with and without diabetes mellitus.

Parameter (Reference Range)	No Diabetes (n = 78)	Diabetes Mellitus (n = 17)	*p*-Value
Fasting glucose (mmol/L)	5.03 (4.54–5.90)	6.28 (5.56–7.61)	<0.0001 *
Fasting insulin (pmol/L)	86.4 (52.6–186.9)	145.8 (94.8–283.2)	0.13
HbA1c (%)	5.4 (5.2–5.7)	6.8 (6.4–7.2)	<0.0001 *
SPINA-GBeta (0.64–3.73 pmol/s)	3.55 (0.26–7.68)	5.09 (2.61–8.76)	0.38
SPINA-GR (1.41–9.00 mol/s)	1.41 (0.69–2.26)	0.61 (0.29–1.35)	0.0057 *
SPINA-DI (4.01–7.65)	5.11 (4.38–6.01)	3.29 (2.43–4.15)	<0.0001 *
HOMA-Beta	207.0 (129.6–439.6)	176.1 (82.3–246.3)	0.11
HOMA-IR (<2.5)	3.2 (1.9–7.1)	7.3 (3.4–9.8)	0.017 *
HOMA-IS (>0.4)	0.3 (0.1–0.5)	0.1 (0.1–0.3)	0.017 *
QUICKI (>0.4)	0.32 (0.29–0.35)	0.29 (0.28–0.32)	0.017 *

* significant result.

**Table 4:** Parameters of insulin–glucose homeostasis in patients with HS and different expressions of the haptoglobin concentration.

Parameter (Reference Range)	Haptoglobin ≤ 209.8 mg/dL (n = 51)	Haptoglobin > 209.8 mg/dL (n = 44)	*p*-Value
Fasting glucose (mmol/L)	5.11 (4.51–5.53)	5.77 (4.81–6.28)	0.043 *
Fasting insulin (pmol/L)	90.0 (53.2–201.0)	100.2 (56.9–186.9)	0.68
HbA1c (%)	5.4 (5.2–5.7)	5.7 (5.3–6.4)	0.0081 *
SPINA-GBeta (0.64–3.73 pmol/s)	3.76 (2.21–8.86)	3.99 (2.43–7.39)	1.00
SPINA-GR (1.41–9.00 mol/s)	1.41 (0.68–2.18)	1.17 (0.55–2.14)	0.30
SPINA-DI (4.01–7.65)	5.11 (4.41–6.00)	4.37 (3.46–5.48)	0.028 *
HOMA-Beta	209.5 (119.4–433.6)	193.9 (127.5–347.0)	0.50
HOMA-IR (<2.5)	3.2 (2.0–7.2)	3.9 (2.1–8.3)	0.43
HOMA-IS (>0.4)	0.3 (0.1–0.5)	0.3 (0.1–0.5)	0.43
QUICKI (>0.4)	0.32 (0.29–0.34)	0.31 (0.28–0.34)	0.43

* significant result.

**Table 5:** Parameters of insulin–glucose homeostasis in patients with HS and with and without antidiabetic treatment.

Parameter (Reference Range)	No Antidiabetic Medication (n = 83)	Antidiabetic Medication (n = 12)	*p*-Value
Fasting glucose (mmol/L)	5.06 (4.56–5.50)	6.75 (6.11–8.03)	<0.0001 *
Fasting insulin (pmol/L)	88.8 (53.1–186.0)	153.0 (65.4–294.3)	0.28
HbA1c (%)	5.5 (5.2–5.8)	7.1 (6.8–8.1)	<0.0001 *
SPINA-GBeta (0.64–3.73 pmol/s)	3.70 (2.32–7.67)	5.29 (2.35–8.93)	0.67
SPINA-GR (1.41–9.00 mol/s)	1.38 (0.68–2.19)	0.53 (0.28–1.38)	0.02 *
SPINA-DI (4.01–7.65)	5.05 (4.30–5.99)	2.92 (2.33–3.52)	<0.0001 *
HOMA-Beta	207.9 (134.6–410.6)	139.9 (75.4–207.2)	0.051
HOMA-IR (<2.5)	3.3 (2.0–7.2)	8.7 (3.0–11.6)	0.051
HOMA-IS (>0.4)	0.3 (0.1–0.5)	0.1 (0.1–0.3)	0.051
QUICKI (>0.4)	0.32 (0.29–0.34)	0.29 (0.27–0.32)	0.051
Hurley stage	2.0 (2.0–3.0)	3.0 (2.8–3.0)	0.046 *
SAHS scale	7.0 (5.0–9.0)	8.0 (7.0–9.3)	0.25
Number of exacerbations	0 (0–2)	0 (0–2)	0.89
Affected regions	3.0 (2.0–5.0)	4.5 (4.0–6.0)	0.051
CRP	5.4 (5.0–13.3)	11.4 (7.9–18.4)	0.109
Haptoglobin	197.4 (153.3–237.8)	256.6 (228.8–291.1)	0.003 *

* significant result.

## Data Availability

The original contributions presented in this study are included in the article. Further inquiries can be directed to the corresponding author (N.A.R.).
